# Molecular Mechanisms of Tibetan Medicinal Sea Buckthorn in the Treatment of Pulmonary Diseases: An Integrated Analysis of Network Pharmacology and Transcriptomics

**DOI:** 10.3390/ijms27146222

**Published:** 2026-07-12

**Authors:** Shanfeng Liang, Benjia Qin, Jiayi Li, Shunzhen Yu, Xudong Tang

**Affiliations:** 1 School of Pharmacy, Gansu University of Chinese Medicine, Lanzhou 730000, China; 15865290230@163.com (S.L.); qinbenjia998@163.com (B.Q.); 18434554113@163.com (J.L.); 18297717183@163.com (S.Y.); 2 Shenzhen Research Institute, Lanzhou University, Shenzhen 518107, China

**Keywords:** sea buckthorn, chronic pulmonary disease, network pharmacology, transcriptomics, immune infiltration

## Abstract

Chronic pulmonary diseases, including chronic obstructive pulmonary disease (COPD), idiopathic interstitial pneumonias (IIPs), pulmonary arterial hypertension (PAH), and pulmonary tuberculosis (PTB), are all marked by persistent immune imbalance, inflammation, and tissue injury. Despite the known anti-inflammatory and antioxidant properties of sea buckthorn, its protective mechanisms across these conditions remain poorly defined. We obtained active compounds and predicted targets from TCMSP and SwissTargetPrediction, and integrated disease genes from GeneCards and CTD with transcriptomic evidence from differential expression analysis and WGCNA. Using SVM-RFE, random forest, and LASSO, we identified hub genes and performed enrichment, immune infiltration, molecular docking, and molecular dynamics analyses. Our results showed that quercetin, kaempferol, and isorhamnetin were the core compounds linked to immune- and inflammation-related targets. The four diseases shared involvement in Toll-like receptor, NOD-like receptor, and related inflammatory pathways, along with recurrent alterations in myeloid cells. Besides, results from molecular docking and molecular dynamics simulations also suggested relatively stable interactions between key compounds and protein targets. Overall, these findings suggest that sea buckthorn may have potential pharmacological relevance across chronic lung diseases. It might do so by modulating common inflammatory signaling pathways and myeloid-related immune responses. At the same time, these findings provide a basis for further experimental validation.

## 1. Introduction

Across the globe, long-lasting lung diseases represent a leading driver of chronic disability and untimely death, continually weighing heavily on public health infrastructures and medical care [1,2,3]. Although COPD, IIPs, PAH, and PTB differ in etiology and clinical presentation, they are linked by several core pathogenic processes. These include sustained inflammatory activation, immune dysregulation, oxidative imbalance, and progressive tissue remodeling. Together, these processes drive disease onset and progression [4,5,6,7,8]. These pathological changes work together to break down lung homeostasis. They harm tissue structure. They also cause a progressive loss of lung function [4,5,8]. Yet, despite advances in diagnostics, clinical management, and drug development, current treatments remain largely limited to controlling symptoms or slowing disease progression. They cannot reverse the complex processes that drive chronic lung injury [1,2,6]. This treatment gap has drawn growing attention to drugs that can act on multiple targets and signaling pathways at the same time.

For a long time, natural medicines have been an important source of pharmacologically active molecules for treating complex chronic diseases [9,10]. Sea buckthorn is a traditional Tibetan medicine. It has long been used to treat respiratory and inflammatory diseases [9]. Phytochemical and pharmacological studies have confirmed that sea buckthorn is rich in various active components. These include flavonoids, phenolic acids, vitamins, and fatty acids. Many of these components possess biological activities such as antioxidant, anti-inflammatory, immunomodulatory, antimicrobial, and tissue-protective effects [10,11,12,13,14,15]. In addition, its beneficial effects have also been reported in several disease contexts, including atopic dermatitis and bone-related diseases [14,15]. In the field of lung disease research, a recent study indicated that sea buckthorn extract may reduce ferroptosis. It does this by clearing reactive oxygen species and regulating the p53/MAPK signaling axis. As a result, it alleviates COPD [13]. These findings suggest that sea buckthorn has potential value for treating chronic lung diseases. Such diseases are marked by immune-inflammatory imbalance and tissue damage [13]. However, most existing studies focus on single compounds or isolated signaling pathways. There is still a lack of system-level evaluations that combine disease omics data with pharmacological predictions [10,11,12,13].

To address the above limitations, network pharmacology provides an effective framework. It helps to explain the “multi-component, multi-target” action features of herbs [16]. However, relying solely on public databases for predictions often fails to capture the biological heterogeneity and molecular complexity of human diseases [16,17]. In contrast, transcriptomic analysis can directly reveal disease-related molecular disturbances [18,19]. WGCNA can identify co-expressed gene modules. These modules contain richer biological information than single differentially expressed genes [19]. On this basis, machine learning methods can be introduced. These include least absolute shrinkage and selection operator (LASSO), random forest (RF), and support vector machine-recursive feature elimination (SVM-RFE). Such methods further enhance the ability to screen for robust biomarkers from high-dimensional data [17,20].Moreover, gene set enrichment analysis (GSEA) and gene set variation analysis (GSVA) offer complementary perspectives. They help interpret biological changes at the pathway level [21,22]. Furthermore, structural methods like molecular docking and molecular dynamics (MD) simulations can be used. These methods assess the feasibility and stability of compound-target interactions [23,24]. By integrating the above approaches, a more comprehensive research strategy can be built. This strategy goes beyond simply relying on traditional network pharmacology.

**Figure 1:**
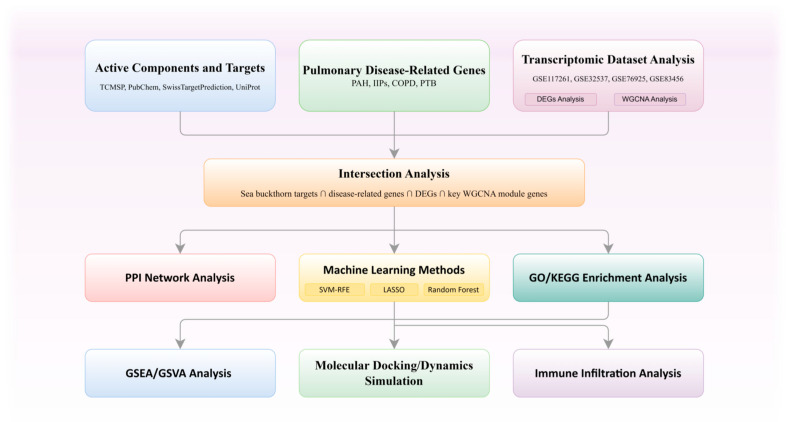
Experimental design.

We adopted an integrative systems pharmacology strategy to investigate how sea buckthorn might act in COPD, IIPs, PAH, and PTB. We retrieved candidate compounds and putative targets from TCMSP and SwissTargetPrediction, and obtained disease-associated genes from GeneCards and CTD. These data were then integrated with differential expression analysis and WGCNA, after which machine learning-based screening was used to pinpoint candidate hub genes. Subsequently, we carried out functional enrichment, immune infiltration analysis, molecular docking, and MD simulations to characterize the pharmacological actions of sea buckthorn at the molecular, pathway, and structural levels. By combining pharmacological prediction, transcriptomic evidence, and computational validation, we aimed to provide a systems-level interpretation of sea buckthorn’s effects in these chronic pulmonary diseases. Figure 1 presents the workflow.

## 2. Results

### 2.1. Candidate Target Identification and Network Features

**Figure 2:**
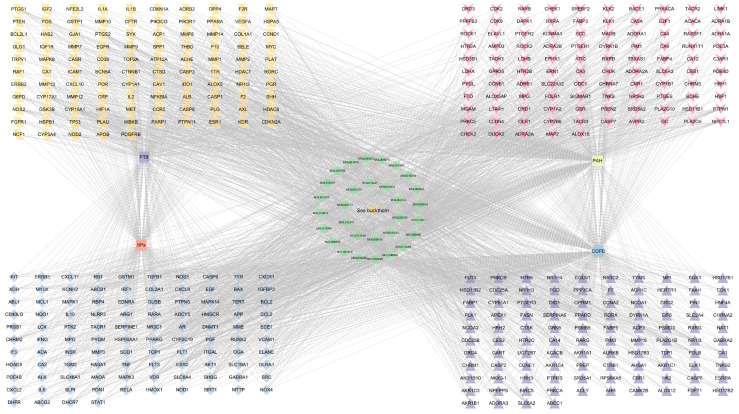
Compound–target–disease regulatory network of the core active components of *Hippophae rhamnoides*.

By integrating data from TCMSP and SwissTargetPrediction, 33 major bioactive compounds of sea buckthorn and 477 corresponding putative targets were obtained (Supplementary Table S1). Disease-related genes for COPD, IIPs, PAH, and PTB were then collected from GeneCards and CTD. After merging the datasets and removing duplicate entries, 413 overlapping targets were identified for COPD, 202 for IIPs, 354 for PAH, and 211 for PTB when intersected with the sea buckthorn target set (Supplementary Table S2). The shared genes were subsequently submitted to the STRING database for protein–protein interaction (PPI) network analysis (Figure S1), while the compound–target–disease network was generated using Cytoscape. Network topology analysis showed that quercetin, kaempferol, isorhamnetin, and pelargonidin had comparatively high degree values, indicating that these flavonoids may represent the main active constituents contributing to the multi-target actions of sea buckthorn (Figure 2; Table S3). We further predicted the physicochemical features, drug likeness indices and pharmacokinetic characteristics of the three key compounds using SwissADME. Complete parameters are compiled in Table S9.

### 2.2. Differential Expression Analysis and Identification of WGCNA Modules

**Figure 3:**
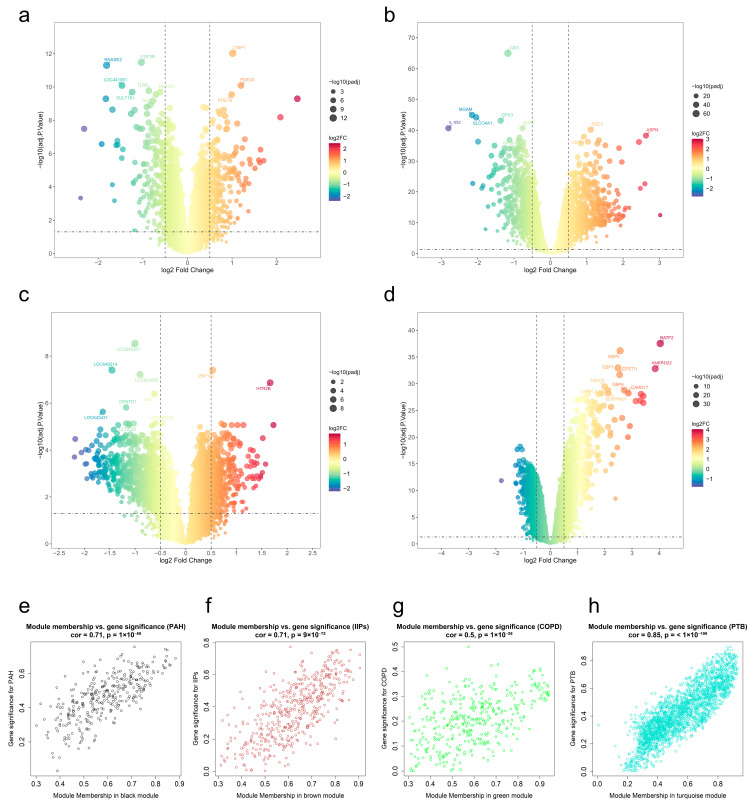
DEG analysis and WGCNA. (**a**) Volcano plot of DEGs for the GSE117261. (**b**) Volcano plot of DEGs for the GSE32537. (**c**) Volcano plot of DEGs for the GSE76925. (**d**) Volcano diagram of DEGs for the GSE83456. (**e**–**h**) Module Membership versus Gene Significance Scatter Plot.

After normalization, the GEO datasets were screened using the predefined thresholds to detect genes with significant expression changes. Their distribution is further illustrated by volcano plots in Figure 3. Subsequently, WGCNA was performed to uncover co-expression modules linked to disease characteristics (Figure S2a–p). Soft-thresholding powers were selected according to the scale-free topology criterion. Stable modules were obtained by dynamic tree cutting and module merging. Figure 3e–h show their relationships with clinical traits. By integrating disease-related genes, differentially expressed genes (DEGs), genes from key WGCNA modules, and sea buckthorn targets, the final sets of potential therapeutic targets were obtained for each disease (Table S2). To further clarify the consistency between transcriptomics-derived disease genes and network pharmacology-predicted sea buckthorn targets, the overlap statistics for DEGs, WGCNA genes, and their intersections were summarized in Table S10.

### 2.3. Functional Enrichment of Potential Therapeutic Targets

**Figure 4:**
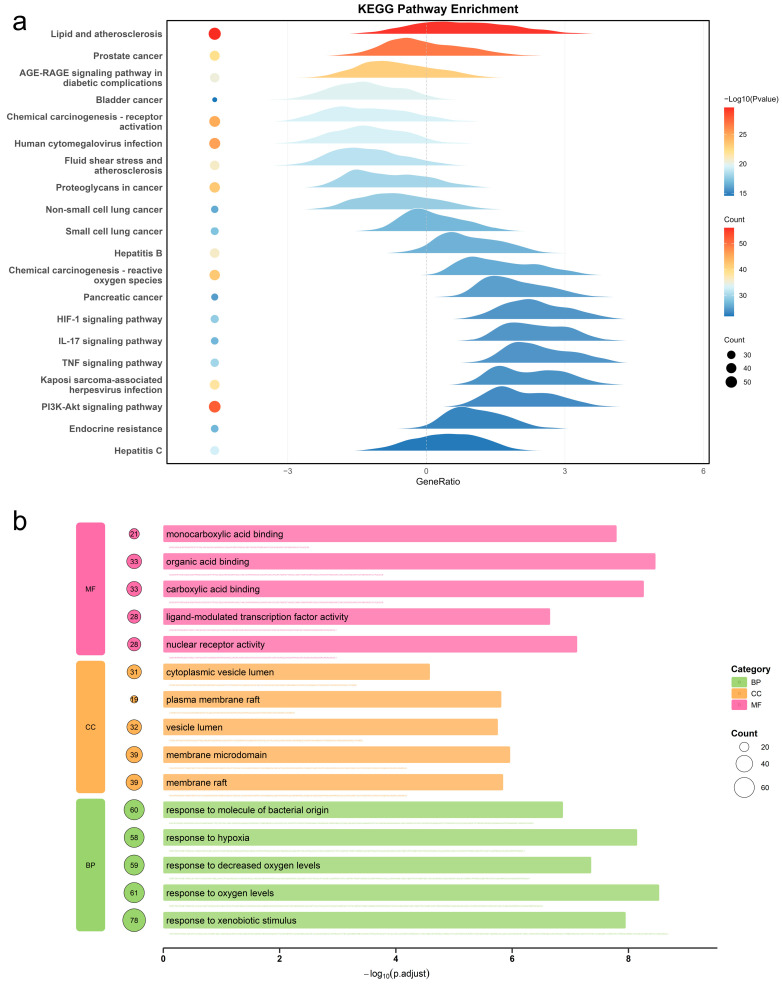
GO and KEGG analyses for COPD. (**a**) Ridge plot showing KEGG pathways. (**b**) Horizontal bar plot showing GO terms.

GO and KEGG analyses were applied to the identified potential targets. Figure 4 and Supplementary Table S6 present the relevant functions and pathways. Across all four pulmonary diseases, the enriched terms were mainly related to immune regulation, inflammatory activity, stress responses, and remodeling of the local tissue microenvironment. In COPD, GO biological process enrichment highlighted responses to xenobiotic stimulation, oxygen variation, and hypoxic stress, suggesting possible involvement of oxidative stress and environmental exposure. In the Cellular Component category, membrane raft, membrane microdomain, and vesicle lumen showed high enrichment. These results suggest roles in membrane signaling and secretion. Molecular function terms were mainly associated with metalloendopeptidase activity and serine-type endopeptidase activity, indicating potential roles in extracellular matrix degradation and proteolytic remodeling. Although IIPs, PAH, and PTB showed comparable enrichment trends, each disease also retained its own specific features. KEGG analysis consistently identified a series of immune- and inflammation-related pathways, including Toll-like receptor signaling, NOD-like receptor signaling, cytokine-cytokine receptor interaction, PI3K-Akt signaling, NF-kappa B signaling, T cell receptor signaling, and B cell receptor signaling.

### 2.4. Hub Gene Identification by Machine Learning

**Figure 5:**
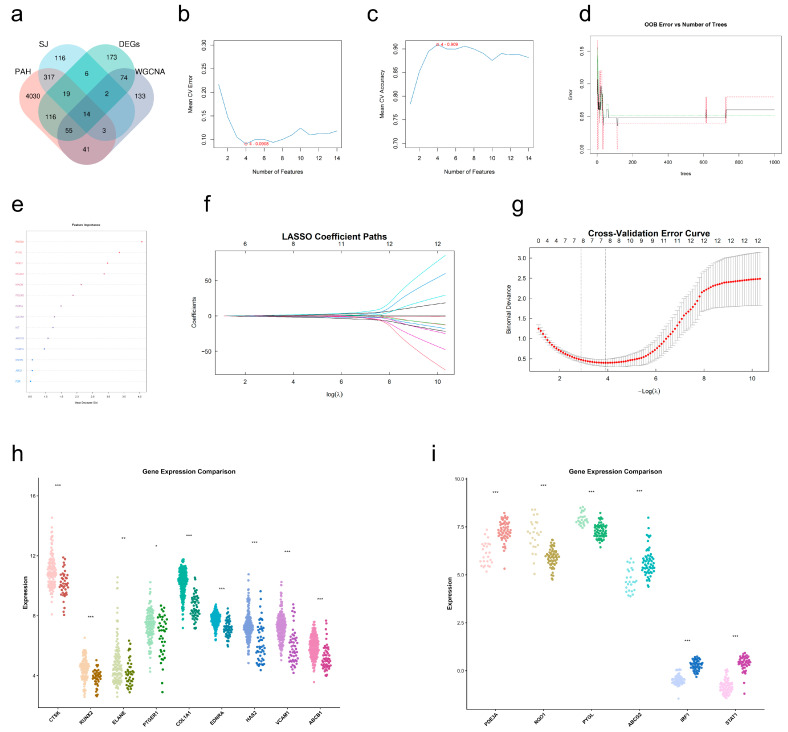
Hub gene screening via machine learning algorithms and expression pattern validation for PAH. (**a**) Venn diagram. (**b**) Cross-validation error curve. (**c**) Cross-validation accuracy curve. (**d**) OOB error curve. (**e**) Feature importance plot. (**f**) LASSO coefficient paths. (**g**) LASSO cross-validation error curve. (**h**,**i**) Bee swarm plots. Colors indicate different genes; within each gene, the lighter shade represents the control group and the darker shade represents the PAH group. * *p* < 0.05, ** *p* < 0.01, *** *p* < 0.001.

Hub genes were screened using nested stratified five-fold cross-validation combined with SVM-RFE, RF, and LASSO. Using the potential therapeutic targets previously identified in PAH as input features for machine learning analysis (Figure 5a), SVM-RFE retained PDE3A, NQO1, PYGL, and ABCG2 (Figure 5b,c). RF ranked PDE3A, PYGL, NQO1, MGAM, MAOB, PDE4D, RORA, CXCR1, KIT, and ABCG2 among the most important variables (Figure 5d,e). LASSO selected ABCG2, NQO1, PDE3A, FABP4, MGAM, PYGL, MAOB, and PDE4D (Figure 5f,g). Overlap analysis among the three methods identified PDE3A, NQO1, PYGL, and ABCG2 as the hub genes in PAH. Using the same analytical strategy, CTSK, RUNX2, ELANE, and PTGER1 were identified in COPD; ABCB1, COL1A1, EDNRA, HAS2, and VCAM1 were identified in IIPs; and STAT1 together with IRF1 were identified in PTB (Table S7). These hub genes showed marked expression differences between disease and control groups and displayed distinct intra-disease correlation patterns (Figure 5h,i).

### 2.5. Integrated Identification of Shared Pathways Across Diseases

**Figure 6:**
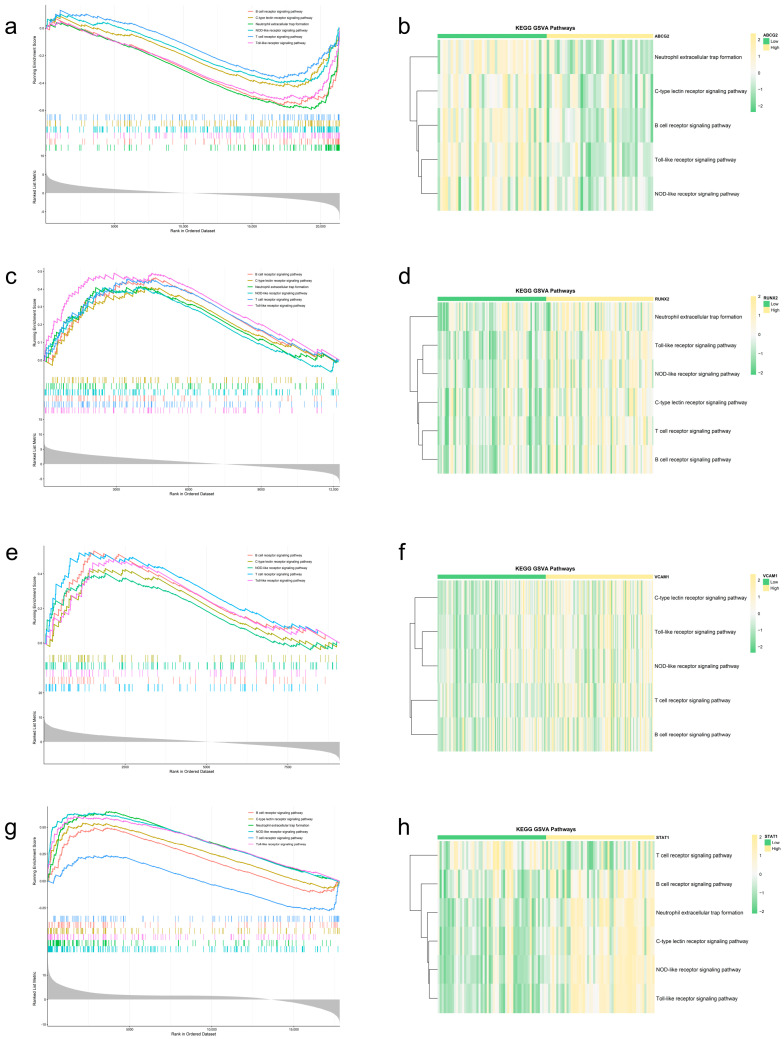
GSEA and GSVA of Pathways. (**a**,**c**,**e**,**g**) GSEA enrichment plots for six immune pathways in PAH, IIPs, COPD, and PTB; (**b**,**d**,**f**,**h**) GSVA enrichment heatmaps for these pathways in the respective disease groups.

To identify robust pathways shared by the four diseases, KEGG enrichment, GSEA, and GSVA were integrated within a two-step framework. First, significantly enriched KEGG pathways common to COPD, IIPs, PAH, and PTB were intersected to define shared disease-related pathways. Next, GSEA and GSVA were performed on the basis of disease-specific hub genes, and the significant pathways obtained from the two approaches were merged after deduplication. The overlap between these results yielded the final set of shared pathways (Figure 6a–h). Using this strategy, 21 common pathways were identified (Table S8). Among them, six immune-related pathways were repeatedly supported across diseases and analytical methods, with consistent enrichment trends: Toll-like receptor signaling, C-type lectin receptor signaling, T cell receptor signaling, B cell receptor signaling, NOD-like receptor signaling, and neutrophil extracellular trap formation (Table 1). These pathways were therefore regarded as the principal shared immune-inflammatory pathways. The exact normalized enrichment score and false discovery rate values for the Toll-like receptor signaling pathway and NOD-like receptor signaling pathway are provided in Table S11. Representative analyses further reinforced these observations. In PTB, higher expression of STAT1 and IRF1 was closely linked to activation of NOD-like receptor signaling and neutrophil extracellular trap formation, whereas T cell receptor signaling tended to be suppressed in the high-STAT1 subgroup. In PAH, elevated PYGL expression was associated with activation of immune-inflammatory pathways, while high ABCG2 expression showed an opposite trend. In IIPs, increased VCAM1 expression corresponded to broad activation of Toll-like receptor, C-type lectin receptor, T cell receptor, B cell receptor, and NOD-like receptor signaling. In COPD, high RUNX2 expression was accompanied by activation of all six shared immune pathways. Consistent with these observations, quantitative analysis of the NETosis pathway score confirmed significantly higher NETosis activity in the RUNX2-high subgroup of COPD and the STAT1-high subgroup of PTB than in their corresponding low-expression subgroups, with a markedly greater subgroup separation in PTB (Figure S4). GSVA results were largely in agreement with the GSEA findings. Taken together, these results suggest marked pathway-level convergence among the four pulmonary diseases, despite clear differences in their individual hub genes.

### 2.6. Immune Infiltration Characteristics in Pulmonary Diseases

**Figure 7:**
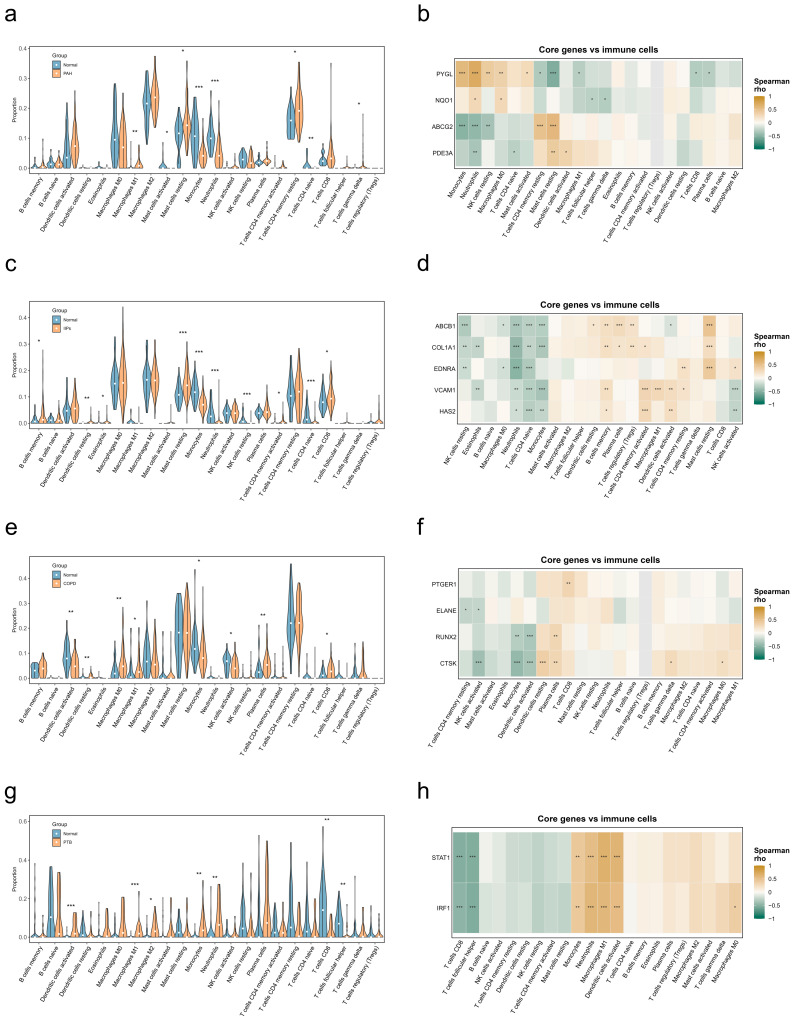
Immune infiltration analysis of PAH, IIPs, COPD, and PTB. (**a**,**b**) Violin plots of immune cell infiltration abundance and Spearman correlation heatmap between core genes and immune cell infiltration levels in PAH. (**c**,**d**) Corresponding analyses for IIPs. (**e**,**f**) Corresponding analyses for COPD. (**g**,**h**) Corresponding analyses for PTB. * *p* < 0.05, ** *p* < 0.01, *** *p* < 0.001.

CIBERSORT was used to estimate immune cell infiltration. Disease and control samples were then compared with the Wilcoxon test, and *p* values were adjusted by the Benjamini–Hochberg method. In COPD, significant changes were observed in activated and resting dendritic cells, M0 and M1 macrophages, monocytes, plasma cells, activated NK cells, and CD8 T cells. CTSK expression was negatively associated with activated NK cells, monocytes, and activated dendritic cells, but positively associated with resting dendritic cells and plasma cells. RUNX2 showed a broadly similar pattern of immune correlations (Figure 7e,f). In IIPs, significant alterations were found in resting mast cells, monocytes, neutrophils, resting NK cells, naive CD4 T cells, resting dendritic cells, memory B cells, eosinophils, activated memory CD4 T cells, and CD8 T cells. The hub genes identified in IIPs were significantly correlated with multiple immune cell populations (Figure 7c,d). In PAH, monocytes and neutrophils were reduced, whereas M1 macrophages and naive CD4 T cells were increased. PYGL expression was positively correlated with monocytes and neutrophils but negatively correlated with resting memory CD4 T cells, while ABCG2 exhibited the reverse relationship (Figure 7a,b). In PTB, activated dendritic cells, M1 macrophages, monocytes, and neutrophils were markedly increased, whereas CD8 T cells, follicular helper T cells, and M2 macrophages were decreased. STAT1 and IRF1 showed positive correlations with pro-inflammatory myeloid cells and negative correlations with CD8 T cells and follicular helper T cells (Figure 7g,h). In particular, STAT1 and IRF1 were positively correlated with M1 macrophages, with Spearman’s rho values of 0.650 and 0.582, respectively (both BH-adjusted *p* < 0.001), further supporting the enrichment of pro-inflammatory myeloid signals in PTB. Overall, recurrent alterations were observed in myeloid immune compartments across all four diseases, particularly in macrophages, monocytes, neutrophils, and dendritic cells, together with accompanying dysregulation of adaptive immune cell subsets.

### 2.7. Molecular Docking

**Figure 8:**
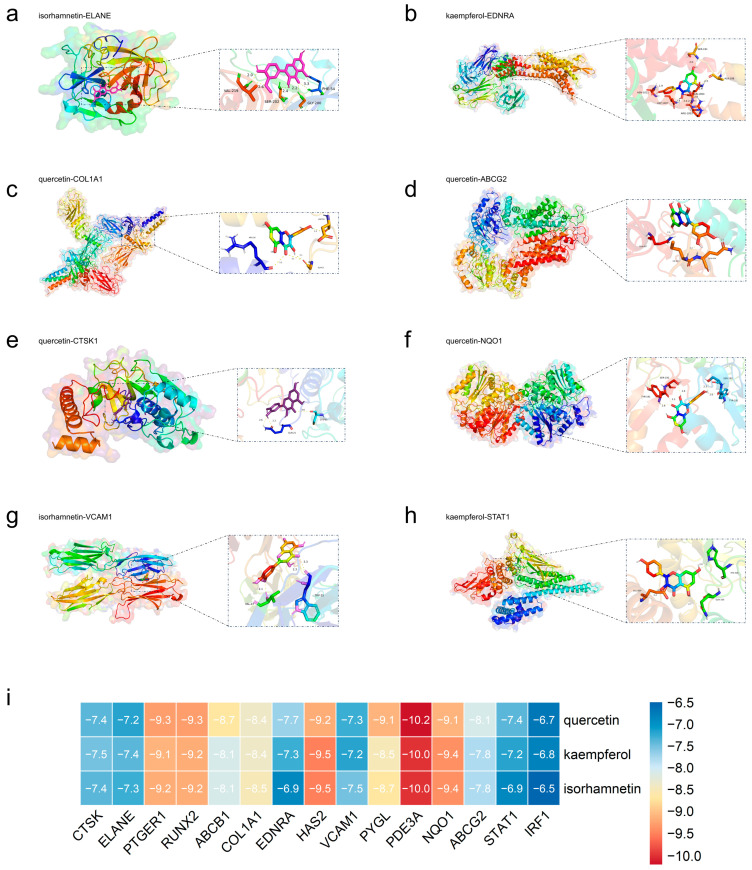
Molecular docking analysis. (**a**–**h**) 3D conformational diagrams of molecular docking. (**i**) Binding free energy heatmap (kcal/mol).

We selected representative hub proteins from different diseases for molecular docking. These included PDE3A, NQO1, PYGL, and ABCG2 for PAH; CTSK, RUNX2, ELANE, and PTGER1 for COPD; ABCB1, COL1A1, VCAM1, EDNRA, and HAS2 for IIPs; and STAT1 and IRF1 for PTB. All protein structures were obtained from the Protein Data Bank (PDB) database. The key active compounds identified through network analysis served as ligands. The docking results showed that most ligand-protein pairs exhibited good binding affinities. This suggested they may form stable interactions (Figure 8i). Representative docking conformations are shown in Figure 8a–h.

### 2.8. Molecular Dynamics Simulation

**Figure 9:**
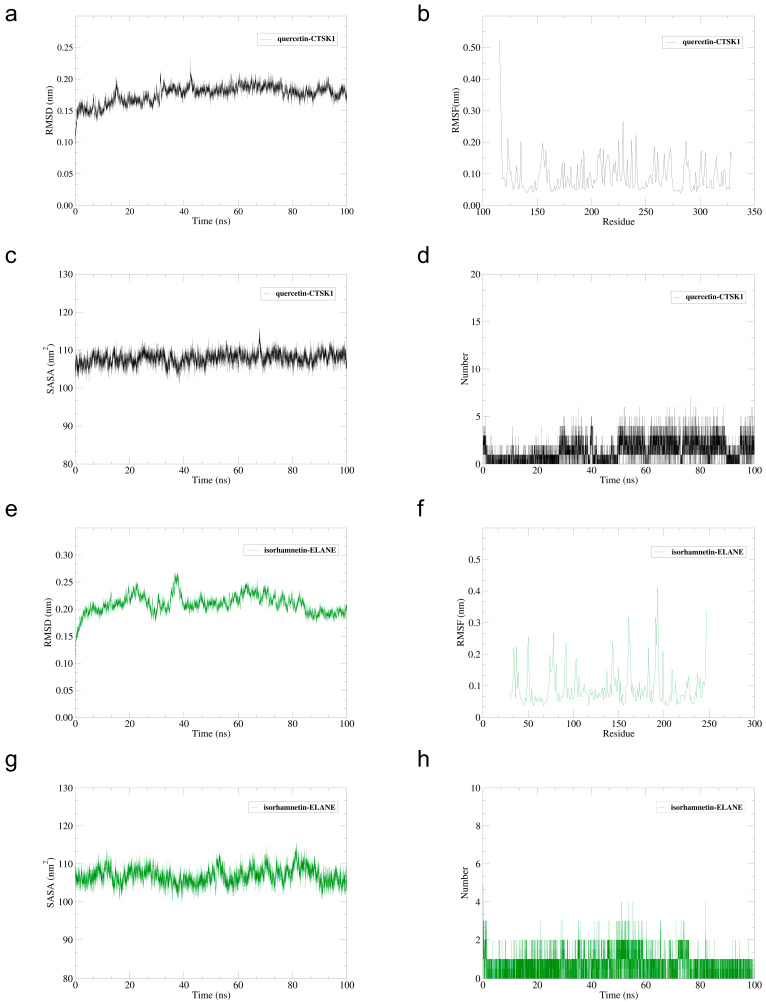
MD simulations of quercetin-CTSK and isorhamnetin-ELANE complexes. (**a**–**d**) RMSD, RMSF, SASA, and hydrogen bond profiles of the quercetin–CTSK1 complex, respectively; (**e**–**h**) RMSD, RMSF, SASA, and hydrogen bond profiles of the isorhamnetin–ELANE complex, respectively.

**Figure 10:**
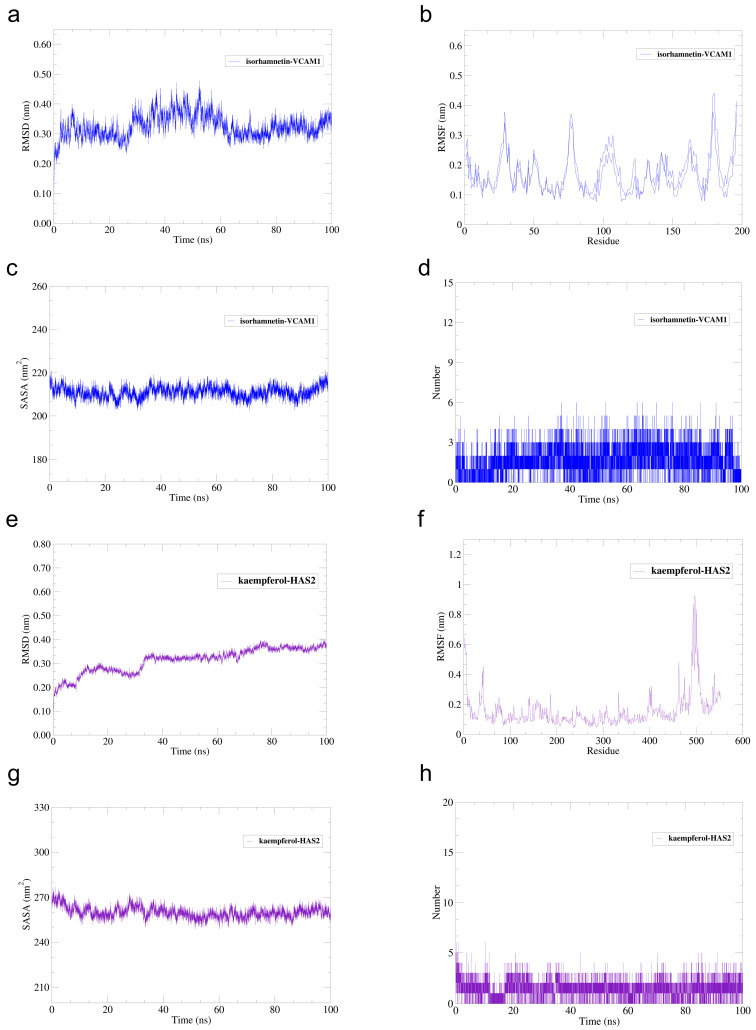
MD simulations of isorhamnetin-VCAM1 and kaempferol-HAS2 complexes. (**a**–**d**) RMSD, RMSF, SASA, and hydrogen bond profiles of the isorhamnetin–VCAM1 complex, respectively; (**e**–**h**) RMSD, RMSF, SASA, and hydrogen bond profiles of the kaempferol–HAS2 complex, respectively.

**Figure 11:**
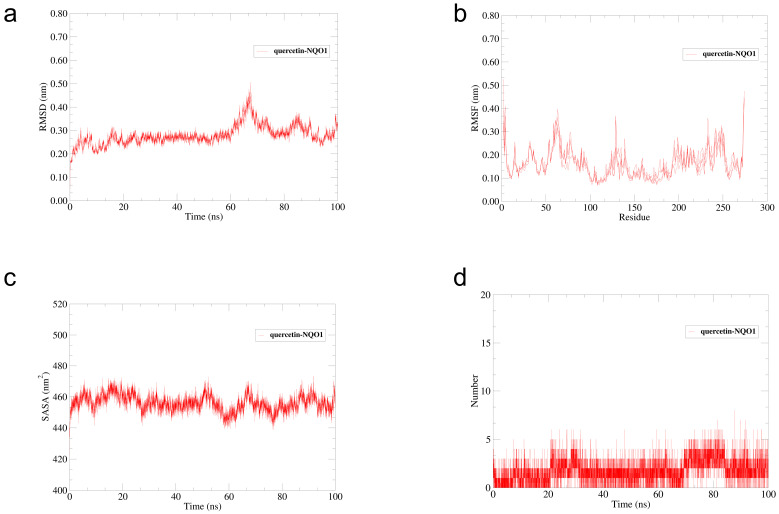
MD simulations of quercetin-NQO1 complex. (**a**–**d**) RMSD, RMSF, SASA, and hydrogen bond profiles of the quercetin–NQO1 complex, respectively.

To assess the dynamic stability of the selected docked models, five systems were analyzed over 100 ns. The simulated systems were quercetin–CTSK (Figure 9a–d), isorhamnetin–ELANE (Figure 9e–h), isorhamnetin–VCAM1 (Figure 10a–d), kaempferol–HAS2 (Figure 10e–h), and quercetin–NQO1 (Figure 11a–d). Throughout the simulations, no ligand dissociation occurred, and all complexes remained globally stable along the trajectories. According to RMSD analysis, the quercetin–CTSK and isorhamnetin–ELANE systems equilibrated at around 15 ns and thereafter maintained relatively stable conformations. The mean RMSD values over the 100 ns simulations were 1.76 ± 0.14 Å for the quercetin–CTSK complex and 2.09 ± 0.17 Å for the isorhamnetin–ELANE complex (mean ± SD). No obvious transient structural instability events were observed in these two systems. The minor short-lived fluctuations in RMSD were considered to represent local conformational adaptations rather than persistent global destabilization. The isorhamnetin–VCAM1 and quercetin–NQO1 complexes were observed to undergo moderate fluctuations; nonetheless, their overall structural stability was preserved. Although the kaempferol–HAS2 complex displayed greater RMSD variation, we did not observe continuous conformational destabilization. These results were corroborated by RMSF and SASA analyses.

The quercetin–CTSK and isorhamnetin–ELANE systems exhibited relatively stable local conformations near the binding regions. Hydrogen bond analysis further revealed persistent intermolecular interactions in all five systems, with quercetin–CTSK, isorhamnetin–ELANE, and isorhamnetin–VCAM1 maintaining relatively stable hydrogen bond profiles over the simulation. Taken together, the MD results support stable binding between major sea buckthorn flavonoids and disease-related hub proteins, lending structural support to the predicted multi-target pharmacological actions of *Hippophae rhamnoides*.

## 3. Discussion

Chronic lung diseases feature ongoing immune-inflammatory activation, oxidative stress, and progressive tissue remodeling. These processes together drive irreversible damage to lung function [25,26,27]. Because such diseases have multifactorial causes, treatments that target a single molecule often show limited benefits. To address this, our study combined network pharmacology, transcriptomic analysis, machine learning, immune infiltration analysis, and molecular simulation. Using these methods, we systematically explored the potential mechanisms of sea buckthorn in multiple lung diseases. The findings indicate that sea buckthorn may exert potential disease-modulating effects through coordinated regulation of immune-inflammatory signaling networks, while also suggesting a broader target profile involving tissue remodeling and myeloid-related injury responses.

In network analysis, quercetin, kaempferol, isorhamnetin, and pelargonidin were identified as major active components. These compounds showed high topological importance in the compound-target network. Although the anti-inflammatory and antioxidant actions of these flavonoids have been widely reported [28,29,30,31,32,33], recent experimental evidence from Liu et al. [13] further supports the potential protective effects of sea buckthorn in COPD. Specifically, their study showed that sea buckthorn extract attenuated COPD-related injury and was associated with inhibition of ferroptosis, suppression of the p53/MAPK pathway, and reduced reactive oxygen species levels. However, our dataset mainly supported the involvement of inflammatory, immune, and tissue-remodeling processes, rather than providing direct gene-level evidence for ferroptosis regulation via the p53/MAPK pathway. Nevertheless, the existing literature suggests that kaempferol may be associated with MAPK signaling [30]. Together, these lines of evidence suggest that the pharmacological effects of sea buckthorn do not stem solely from single-target inhibition, but may involve multi-component and multi-target interactions. In particular, our results point not only to canonical inflammatory pathways, but also to target combinations related to extracellular matrix remodeling, endothelial activation, protease activity, and oxidative stress regulation. Our results further suggest the potential involvement of pathways related to oxidative stress and programmed cell death; however, the present analyses do not provide direct evidence that these processes constitute dominant mechanisms, and further experimental validation is required.

By integrating differential expression analysis, WGCNA, and machine learning algorithms, this study identified multiple hub genes closely related to disease. Most of these genes already have well-established biological links to lung pathology. In fibrotic interstitial lung disease, COL1A1 is involved in fibrosis progression. VCAM1 has been reported as a TGF-β1-inducible gene and is upregulated in idiopathic pulmonary fibrosis. Together, these two genes suggest possible roles in extracellular matrix deposition and endothelial cell activation [34,35]. In pulmonary arterial hypertension, PDE3A is associated with the growth and metabolism of pulmonary artery smooth muscle cells. NQO1 is involved in antioxidant defense mechanisms and redox imbalance in experimental pulmonary arterial hypertension [36,37]. In pulmonary tuberculosis, STAT1 acts as a key mediator of IFN-γ-driven signals. Its activation in alveolar macrophages supports host immune responses against mycobacterial infection [38]. IRF1 participates in interferon-response-related immune regulation and macrophage activation. Moreover, an IRF8/IRF1-dependent transcriptional program is significantly enriched in pulmonary tuberculosis [39]. These genes were consistently identified across multiple analytical strategies. This further highlights their potential biological significance. Notably, some targets highlighted in our integrated analysis, such as CTSK, HAS2, ELANE, VCAM1, and NQO1, suggest that sea buckthorn may influence protease-associated tissue injury, hyaluronan/extracellular matrix remodeling, endothelial adhesion, and redox homeostasis in addition to general inflammatory signaling.

We combined the results from KEGG, GSEA, and GSVA. Six immune-related pathways were recurrently identified across these analyses: the Toll-like receptor signaling pathway, the NOD-like receptor signaling pathway, the T cell receptor signaling pathway, the B cell receptor signaling pathway, the C-type lectin receptor signaling pathway, and the neutrophil extracellular trap (NET) formation pathway. The recurrent enrichment of these pathways across all three methods suggests a broadly consistent involvement of immune-related processes. TLR and NLR pathways act as critical sensors of pathogen- and danger-associated signals and are central to the initiation of innate immune responses [40,41]. Their dysregulated activation contributes to chronic inflammatory and infectious pulmonary disorders [42,43]. TCR and BCR signaling further indicate alterations in adaptive immunity, while CLR signaling participates in antifungal and antimycobacterial immunity [44]. Growing evidence also indicates that excessive NET formation can aggravate tissue injury and amplify inflammation in the lung [45,46]. Taken together, these results suggest that immune-inflammatory regulation may be an important component of the pharmacological effects of sea buckthorn. Importantly, the concomitant involvement of NET formation and disease-relevant targets such as ELANE, together with remodeling-related molecules such as HAS2 and CTSK, suggests that the potential actions of sea buckthorn may extend beyond broad NF-κB/TLR-type anti-inflammatory effects. Nevertheless, the observed pathway convergence is more consistent with overlapping functional patterns than with definitive shared mechanisms across all datasets.

The immune infiltration results were consistent with these findings. We observed alterations in macrophages, monocytes, neutrophils, dendritic cells, and lymphocyte subsets across all four pulmonary diseases. Correlations between hub genes and immune cell populations suggest that the identified targets are involved in regulating immune cell recruitment and activation. In pulmonary tuberculosis, the positive correlations of STAT1 and IRF1 with Macrophages M1 suggest an interferon-associated inflammatory program in the immune microenvironment [47,48]. However, this pattern may reflect either antimicrobial host defense or sustained inflammation associated with immune dysregulation and tissue injury. Given the correlative nature of this transcriptomic analysis, these findings support interferon-linked macrophage activation but do not establish pathological interferon overactivation. Similarly, in chronic obstructive pulmonary disease, ELANE showed a weak positive correlation with neutrophils (Spearman’s rho = 0.208; raw *p* = 0.016; BH-adjusted *p* = 0.076). Although this association did not remain statistically significant after multiple-testing correction, its positive direction is biologically plausible, given that ELANE encodes neutrophil elastase, a key neutrophil-derived protease involved in airway inflammatory injury [49]. Together, these observations further support a potential link between transcriptomic changes and immune microenvironment remodeling, especially myeloid-cell-associated inflammatory injury.

Molecular docking and molecular dynamics simulations provided structural evidence. They confirmed the interactions between major flavonoids from sea buckthorn and core target proteins. Quercetin, kaempferol, and isorhamnetin all showed good binding affinity for disease-related targets. Among these targets, HAS2 may be biologically relevant in PAH because extracellular matrix remodeling is a key feature of pulmonary vascular remodeling, and HAS2 participates in hyaluronan synthesis associated with this process. A 100-nanosecond molecular dynamics simulation revealed that representative ligand-protein complexes maintained relatively stable conformations under physiological conditions. Among them, the quercetin-CTSK and isorhamnetin-ELANE complexes exhibited stable root-mean-square deviation (RMSD) and hydrogen bond profiles throughout the simulation. This suggested relatively stable ligand-target associations under the simulated conditions. However, docking scores and MD stability do not by themselves demonstrate target engagement, pharmacodynamic activity, or therapeutic efficacy in vivo. They only support the structural feasibility of ligand–target binding under simulated conditions. These computational findings require further experimental validation. Nevertheless, they provide computational support for a possible multi-target pharmacological basis of sea buckthorn, including possible effects on processes related to CTSK, ELANE, and HAS2.

Although we have found that these diseases share common immune-inflammatory mechanisms, each also has its own distinct features. COPD is more closely linked to oxidative stress and protease pathways. This is consistent with its chronic airway injury caused by environmental exposure [26,50]. In idiopathic interstitial pneumonia, the most prominent characteristic is the significant enrichment of processes related to extracellular matrix remodeling and fibrosis [51]. Pulmonary arterial hypertension mainly involves inflammation-driven vascular remodeling [52]. Tuberculosis is marked by strong activation of interferon and inflammatory immune signals [47,48]. These differences suggest that sea buckthorn may regulate shared inflammatory pathways. However, its biological effects may differ depending on the disease context. Thus, rather than acting only as a generic anti-inflammatory natural product, sea buckthorn may show a pulmonary-relevant target pattern involving neutrophil-associated injury, extracellular matrix/hyaluronan remodeling, vascular adhesion, and oxidative stress defense. Overall, our findings suggest shared pathway-level and immune-inflammatory patterns across these diseases, together with several more specific target features of potential relevance to sea buckthorn, rather than a clearly selective pharmacological action of sea buckthorn.

To clarify the relationship between transcriptomics-derived disease genes and network pharmacology-predicted targets, we compared the 477 predicted sea buckthorn targets with the DEG sets and key WGCNA module genes for each disease. The overlap was modest overall: 32/1246 DEGs and 15/385 WGCNA genes in COPD, 71/1430 and 22/463 in IIPs, 41/459 and 19/322 in PAH, and 45/1615 and 69/2260 in PTB, with triple intersections of 10, 10, 16, and 39 genes, respectively. This partial concordance likely reflects the distinct nature of the two data sources, as transcriptomics/WGCNA capture disease-associated alterations, whereas network pharmacology predicts putative compound targets from available databases. Therefore, the limited overlap should be acknowledged as a methodological limitation and may indicate indirect effects, pathway-level regulation, or incomplete target coverage in current databases.

This study has some limitations. Although we combined multiple computational methods to improve the robustness of our results, the conclusions are still mainly based on bioinformatics predictions. In addition, the exclusive use of the TCMSP database and the default TCMSP screening thresholds (OB ≥ 30% and DL ≥ 0.18) may have excluded potentially relevant natural compounds, particularly flavonoids with low predicted oral bioavailability, as well as other minor constituents, thereby limiting the completeness of compound screening. This may also reduce the ability of the current approach to capture the potential synergistic effects of the whole sea buckthorn extract. Further experimental validation in cell and animal models is therefore needed. Moreover, the major active compounds identified in this study are mainly flavonoids, whose limited oral bioavailability may hinder their ability to reach effective concentrations in lung tissue. Therefore, the in vivo relevance of the predicted targets and pathways remains uncertain and requires further pharmacokinetic and experimental validation. Future translation may require improved delivery strategies, such as nanoparticles, liposomal formulations, or inhalable preparations, to enhance lung exposure and therapeutic potential.

## 4. Materials and Methods

### 4.1. Component Acquisition and Target Prediction

We collected chemical components of sea buckthorn from the TCMSP database (accessed on 18 February 2026) [53]. Compounds were retained as candidate bioactive constituents when oral bioavailability was at least 30% and drug-likeness was no less than 0.18. Potential targets from TCMSP were further checked in Universal Protein Knowledgebase (accessed on 6 January 2026) [54]. The species was limited to humans. Official gene symbols were extracted from the matched records. To broaden target coverage, we first obtained the SMILES strings of the selected compounds from PubChem (accessed on 10 January 2026). Next, we used SwissTargetPrediction 2019 to predict their possible human targets [55,56]. Target lists obtained from TCMSP and SwissTargetPrediction were merged, and duplicate entries were removed before subsequent analysis.

### 4.2. Collection of Genes Related to Pulmonary Diseases

Disease-associated genes for COPD, IIPs, PAH, and PTB were gathered from GeneCards (accessed on 12 January 2026) and the Comparative Toxicogenomics Database (accessed on 12 January 2026) [57,58]. In GeneCards, genes with a relevance score of 0.5 or higher were included. In CTD, the top 150 genes ranked by inference score were selected for each disease. After combining the two resources and removing redundant entries, disease-specific gene sets were generated for later analyses.

### 4.3. Definition of Candidate Therapeutic Targets

For each pulmonary disease, we compared the predicted targets of *Hippophae rhamnoides* against the corresponding disease gene sets. The shared genes were considered potential therapeutic targets of sea buckthorn. The shared genes were considered candidate therapeutic targets and were carried forward for network and functional analyses.

### 4.4. Construction of PPI Networks and Functional Enrichment Analysis

The candidate therapeutic targets were submitted to the STRING database (version 12.0) to establish PPI networks, with the species parameter set to Homo sapiens [59]. Functional annotation was performed in R using the clusterProfiler package (version 4.18.4) to conduct GO and KEGG enrichment analyses [60,61]. *p* values were adjusted with the Benjamini–Hochberg method, and adjusted *p* values below 0.05 were regarded as statistically significant.

### 4.5. Retrieval of Transcriptomic Datasets

For COPD, IIPs, PAH, and PTB, the corresponding gene expression data were collected from the GEO repository (accessed on 15 January 2026). After considering sample size, data quality, and platform comparability, GSE76925, GSE32537, GSE117261, and GSE83456 were selected as the primary datasets for downstream transcriptomic analyses [62].

### 4.6. Differential Expression Analysis and WGCNA

Differential expression analysis of GSE76925, GSE32537, GSE117261, and GSE83456 was conducted using the limma package (version 3.66.0) in R [63]. Genes meeting the criteria of |log2 fold change| ≥ 0.5 and adjusted *p* < 0.05 were defined as DEGs. Multiple testing was corrected using the Benjamini–Hochberg procedure. WGCNA was then applied to identify modules associated with disease traits [64]. For each dataset, the 5000 genes with the highest median absolute deviation values were used to construct the network. Following sample clustering, the soft-thresholding power was chosen according to the scale-free topology criterion. The adjacency matrix was subsequently converted into a topological overlap matrix, and gene modules were identified by hierarchical clustering combined with dynamic tree cutting. Modules showing the strongest association with disease phenotype were regarded as key modules. Genes located at the intersection of disease-associated genes, DEGs, key module genes, and sea buckthorn targets were defined as potential therapeutic targets. In addition, overlap statistics between sea buckthorn targets and the DEG/WGCNA-derived gene sets were summarized for interpretive comparison.

### 4.7. Establishment of the Compound–Target–Disease Network

To clarify the relationships among the identified active compounds, overlapping targets, and disease nodes, we used Cytoscape v3.10.3 to construct a compound–target–disease network [65]. where nodes represent compounds, targets, and disease, and edges represent their interactions. Degree value was used as the main topological index for network evaluation, and compounds ranked among the top 10 by degree were treated as key active constituents.

### 4.8. Machine Learning

Hub genes were screened using three machine learning approaches: SVM-RFE, RF, and LASSO. These analyses were implemented in R with the e1071 (version 1.7.17), randomForest (version 4.7.1.2), and glmnet (version 4.1.10) packages, respectively [66,67,68]. To obtain more reliable evaluation results and reduce the likelihood of overfitting, we applied nested stratified cross-validation across five folds. In this procedure, parameter optimization together with feature selection was conducted in the inner loop, while the outer loop served to evaluate the model [69]. The predictive ability of the model was subsequently examined using ROC and PR curves. For datasets with class imbalance, class weights were introduced when required. Genes simultaneously retained by all three algorithms were considered hub genes.

### 4.9. Integrated Pathway Analysis Using KEGG, GSEA, and GSVA

To investigate the biological functions of hub genes from multiple perspectives, KEGG enrichment, GSEA, and GSVA were performed in R using clusterProfiler (version 4.18.4), limma (version 3.66.0), and GSVA (version 2.4.8) [61,70,71]. Gene symbols were converted to ENTREZ IDs using org.Hs.eg.db (version 3.22.0), and KEGG pathway sets were used as the reference for pathway interpretation. In GSEA, samples were categorized into high- and low-expression groups according to the median expression level of each hub gene, and preranked enrichment analysis was performed based on limma t statistics [70]. GSVA was then applied to estimate pathway activity scores at the sample level [71]. GSVA score differences were analyzed using limma, and correlations with hub-gene expression were evaluated by Spearman analysis with Benjamini–Hochberg correction. Pathways supported by KEGG enrichment and showing consistent trends in both GSEA and GSVA were considered high-confidence candidate pathways.

### 4.10. Immune Cell Infiltration Analysis

Immune infiltration patterns in GSE76925, GSE32537, GSE117261, and GSE83456 were estimated with CIBERSORT [72]. Only samples with CIBERSORT *p* < 0.05 were retained for further analysis. Associations between hub gene expression and immune cell abundance were evaluated using Spearman correlation, followed by Benjamini–Hochberg correction for multiple comparisons.

### 4.11. Molecular Docking

Three-dimensional structures of the target proteins were downloaded from the PDB [73], and ligand structures were retrieved from PubChem [55]. Protein structures were prepared in PyMOL (version 3.1.3.1) by removing water molecules and co-crystallized small molecules before docking. Ligand structures were optimized in Chem3D (version 23.1.1.3) using the MM2 force field for energy minimization. Then, AutoDock Vina (version 1.1.2) [74] was used for the docking study. The conformation with the minimum binding energy was regarded as the optimal pose.

### 4.12. Molecular Dynamics Simulation

We used GROMACS 2020.6 [75] to perform MD simulations on selected protein–ligand complexes. In the simulations, the protein was represented using the AMBER99SB-ILDN model, whereas the ligand was parameterized with a force field compatible with the AMBER framework [76]. A cubic solvent box was built for every complex. The protein was kept 1.0 nm away from the box edge. Counterions were added to neutralize the charge. Energy minimization was then carried out. The steepest descent method was run for 50,000 iterations. Following minimization, a 100-ps NVT equilibration step was applied. The system then underwent a 100-ps NPT equilibration step. Next came a 100-ns production run at 300 K and 1.0 bar with periodic boundary conditions.

## 5. Conclusions

We integrated network pharmacology, transcriptomic profiling, machine learning, immune infiltration analysis, and molecular simulation to explore how sea buckthorn might act in COPD, IIPs, PAH, and PTB. Our findings suggest that sea buckthorn may be involved in shared immune-inflammatory pathways, including Toll-like receptor signaling, NOD-like receptor signaling, T/B cell receptor signaling, and neutrophil extracellular trap formation, together with possible modulation of myeloid immune responses. Quercetin, kaempferol, and isorhamnetin were identified as the major active flavonoids with potential interactions with multiple disease-associated proteins. KEGG enrichment, GSEA, and GSVA analyses consistently revealed substantial pathway-level convergence across the four diseases, while molecular docking and MD simulations supported the structural plausibility of representative compound-target interactions. In addition to these common inflammatory pathways, targets such as CTSK, HAS2, ELANE, VCAM1, and NQO1 suggest that sea buckthorn may also influence protease-associated tissue injury, extracellular matrix/hyaluronan remodeling, endothelial activation, and oxidative stress regulation. Taken together, these findings suggest a potential role for sea buckthorn in modulating shared pathogenic pathways across chronic pulmonary disorders, with a target profile extending beyond generic anti-inflammatory effects. Further experimental studies are needed to confirm these predictions.

## Figures and Tables

**Table 1:** Six Shared Immune Pathways.

KEGG ID	Pathway Name
hsa04620	Toll-like receptor signaling pathway
hsa04625	C-type lectin receptor signaling pathway
hsa04660	T cell receptor signaling pathway
hsa04662	B cell receptor signaling pathway
hsa04621	NOD-like receptor signaling pathway
hsa04613	Neutrophil extracellular trap formation

## Data Availability

The original contributions presented in this study are included in the article/Supplementary Materials. Further inquiries can be directed to the corresponding author.

## Supplementary Materials

The following supporting information can be downloaded at: https://www.mdpi.com/article/10.3390/ijms27146222/s1.
