# Supplementary material for: Molecular Mechanisms of Tibetan Medicinal Sea Buckthorn in the Treatment of Pulmonary Diseases: An Integrated Analysis of Network Pharmacology and Transcriptomics
Source: Int J Mol Sci. 2026 Jul 12;27(14):6222. doi: 10.3390/ijms27146222 (PMC13410159; doi:10.3390/ijms27146222)
Supplement: Supplementary file 1 [file ijms-27-06222-s001.zip › Supplementary Figures.pdf]

Supplementary Materials

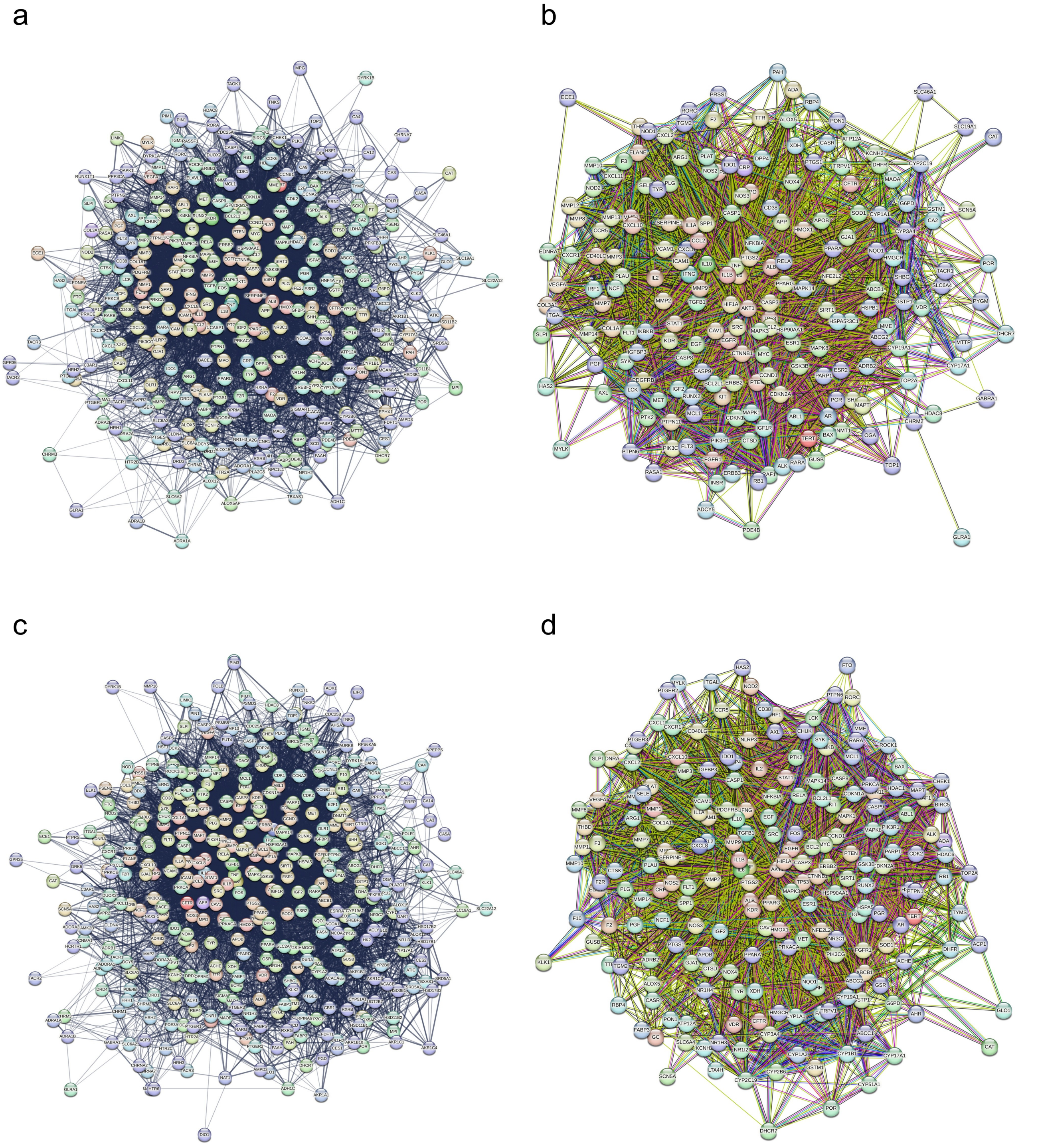

Figure S1. PPI network of putative targets related to sea buckthorn in chronic pulmonary diseases. (a) PAH. (b) IIPs. (c) COPD. (d) PTB.

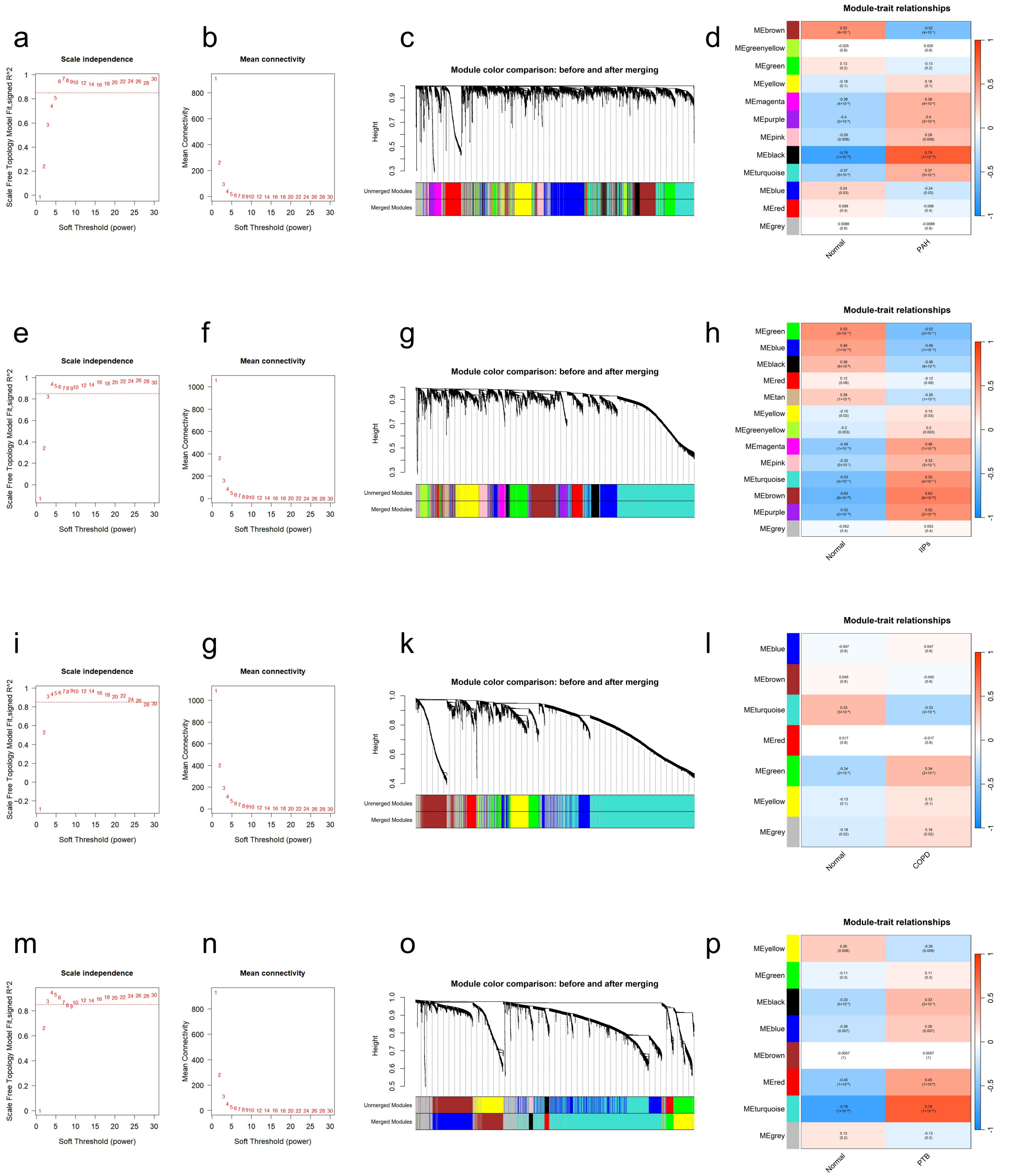

Figure S2. Weighted gene co-expression network analysis (WGCNA) for four pulmonary diseases. (a–d) Results for PAH. (e–h) Results for IIPs. (i–l) Results for COPD. (m–p) Results for PTB. For each disease group: (a, e, i, m) Scale independence analysis for soft-threshold power selection; (b, f, j, n) Mean connectivity analysis; (c, g, k, o) Gene clustering dendrogram with module color assignment before and after merging; (d, h, l, p) Heatmap of module-trait relationships.

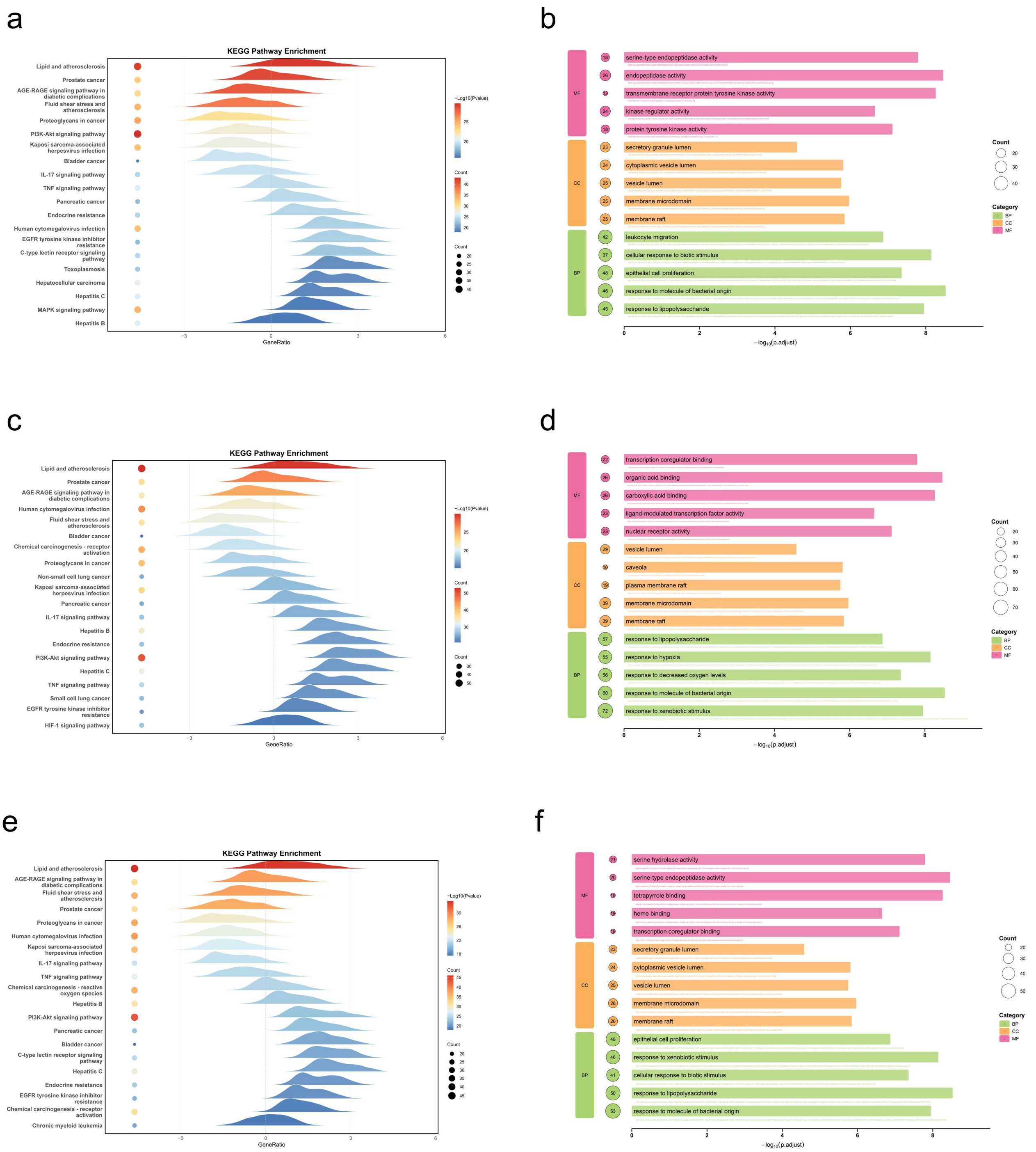

Figure S3. GO and KEGG Analyses. (a,b) KEGG pathway ridge plots and GO term horizontal bar plots for IIPs. (c,d) KEGG pathway ridge plots and GO term horizontal bar plots for PAH. (e,f) KEGG pathway ridge plots and GO term horizontal bar plots for PTB.

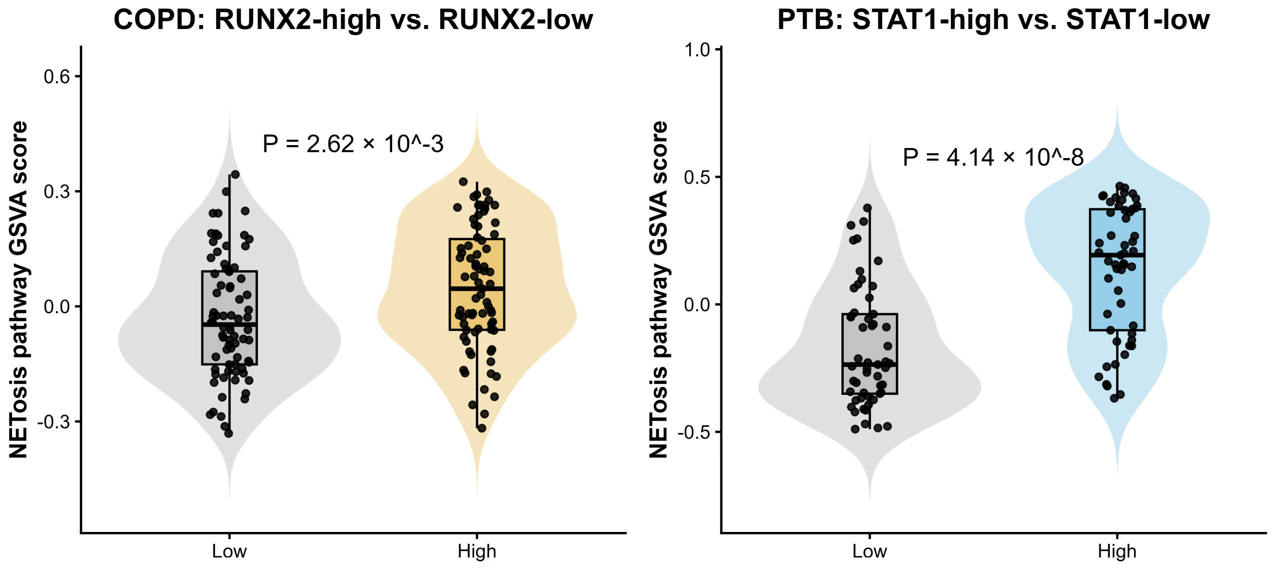

Figure S4. Quantitative comparison of GSVA scores for the KEGG NETosis pathway between RUNX2-high and RUNX2-low subgroups in COPD and between STAT1-high and STAT1-low subgroups in PTB.
